# Noninvasive Blood Glucose Monitoring Systems Using Near-Infrared Technology—A Review

**DOI:** 10.3390/s22134855

**Published:** 2022-06-27

**Authors:** Aminah Hina, Wala Saadeh

**Affiliations:** Department of Electrical Engineering, Lahore University of Management and Sciences, Lahore 54792, Pakistan; aminah.hina@lums.edu.pk

**Keywords:** noninvasive glucose monitoring, Photoplethysmography (PPG), near-infrared (NIR), machine learning (ML) methods

## Abstract

The past few decades have seen ongoing development of continuous glucose monitoring (CGM) systems that are noninvasive and accurately measure blood glucose levels. The conventional finger-prick method, though accurate, is not feasible for use multiple times a day, as it is painful and test strips are expensive. Although minimally invasive and noninvasive CGM systems have been introduced into the market, they are expensive and require finger-prick calibrations. As the diabetes trend is high in low- and middle-income countries, a cost-effective and easy-to-use noninvasive glucose monitoring device is the need of the hour. This review paper briefly discusses the noninvasive glucose measuring technologies and their related research work. The technologies discussed are optical, transdermal, and enzymatic. The paper focuses on Near Infrared (NIR) technology and NIR Photoplethysmography (PPG) for blood glucose prediction. Feature extraction from PPG signals and glucose prediction with machine learning methods are discussed. The review concludes with key points and insights for future development of PPG NIR-based blood glucose monitoring systems.

## 1. Introduction

Diabetes mellitus is one of the most chronic diseases in the world with an annual death rate of 1.5 million. [1]. International Diabetes Federation (IDF) records show that in the year 2021 alone, 6.7 million deaths occurred due to diabetes [2]. Approximately 537 million adults (1 in 10) with ages ranging from 20 to 79 years are living with diabetes worldwide. It is not only chronic, but diabetes is also becoming one of the most globally prevalent diseases with a projection of a 46% increase in patients by the year 2045 [2]. Diabetes is on the rise at an alarming rate, especially in low- and middle-income countries, where every 3 out of 4 adults is suffering from diabetes [2]. One of the main reasons for the increase in diabetes is an increase in obesity and health unawareness. Unhealthy lifestyles, which include consumption of unhealthy foods (junk food, soft drinks, and bakery items) and lack of exercise, majorly contribute to obesity. Due to a lack of knowledge, many people are unaware of their pre-diabetic conditions, resulting in diabetes eventually. Diabetes causes many other serious health issues, which include cardiovascular diseases, renal diseases, nervous system damage, and vision impairment. If blood glucose levels are not properly monitored and controlled, these illnesses can further aggravate, causing organ failures and eventually leading to death.

Insulin is a hormone made by the pancreas, which regulates blood glucose levels (BGL) in the human body. In diabetic patients, either the pancreas are unable to produce enough insulin hormone or the cells in the body are not responding to insulin (insulin resistance). Insufficient insulin production is classified as Type 1 diabetes and is mostly diagnosed in children. Insulin resistance is classified as Type 2 diabetes. Diabetes developed during pregnancy is gestational diabetes [3]. The most diagnosed diabetes in patients worldwide is Type 2 diabetes. Every year sees a huge increase in patients in the diabetic community. Pre-diabetic conditions, gestational diabetes, and impaired glucose tolerance need to be monitored closely to avoid developing diabetes. There is no cure for diabetes, hence effective diabetic management is the only solution for diabetic patients for having a quality life. Effective diabetic management includes treatment by taking artificial insulin, either by shots or medicine, exercise, a careful diet intake, and frequent blood glucose monitoring.

The conventional method for checking BGL is the finger-prick method. As it can be done by the patient, it is called a self-monitoring blood glucose (SMBG) monitoring device. It is an electrochemical-based method in which the finger is pricked to draw small drops of blood on a test strip, and a discrete glucose value is displayed within a few seconds [4]. This method is the golden standard for patients’ blood glucose measurements at home. Despite its high accuracy, this method is still inconvenient for many patients to take measurements several times a day, as finger pricking is slightly painful, and the test strips are expensive and for one-time use only. These reasons make it undesirable for checking BGL several times a day, especially in low-income countries. Limiting to 2–3 measurements a day cannot accurately track glucose variations in blood levels throughout the day. This led to the idea of developing devices that could assist in continuous monitoring without being invasive. In the year 2004, the first commercial continuous glucose monitoring (CGM) device was introduced by Medtronic (San Jose, CA, USA) for patients’ personal use [5]. Later, Dexcon, Inc. and Abbott Diabetes Care (San Diego, CA, USA) launched CGM, targeting the long life span of implanted patches with better accuracy [6,7]. Since then, the industries are working on improving and updating the devices’ accuracy, lifespan, and calibration issues. Smart features have also been introduced in CGM devices graphically, displaying variation in glucose levels on smartphones, and sound indicators for high and low glucose levels [5,8]. Although CGM devices are available in the international market, they still have many challenges to overcome. They are semi or minimally invasive devices which need to be implanted by medical professionals. They are expensive, a bit complex to use, and have low accuracy compared to the invasive method. To attract the global market and mass consumers, CGM devices need to improve in accuracy, cost, and technology. The past two decades have also seen many research trends in the development of optical devices for glucose monitoring, aiming to be cost-effective and noninvasive. Many research works have shown promising results using different technologies [9,10].

In summary, minimally invasive and non-invasive glucose monitoring technologies have become an interesting and emerging research field. The researchers aim to offer a solution for continuous glucose monitoring with minimal pain or painless methods, with the integration of smart monitoring. This review paper will be discussing noninvasive BGL monitoring technologies with a particular focus on research work in NIR technology. Section 2 will discuss some of the research work done in noninvasive glucose monitoring using different noninvasive techniques. Section 3 briefly describes NIR spectrometry. Section 4 discusses research works using the NIR Technique for BGL monitoring, with a particular focus on feature extraction from Photoplethysmography (PPG) signals. Section 5 concludes the discussion with key points and future directions.

## 2. Noninvasive Glucose Sensing Methods

**Figure 1:**
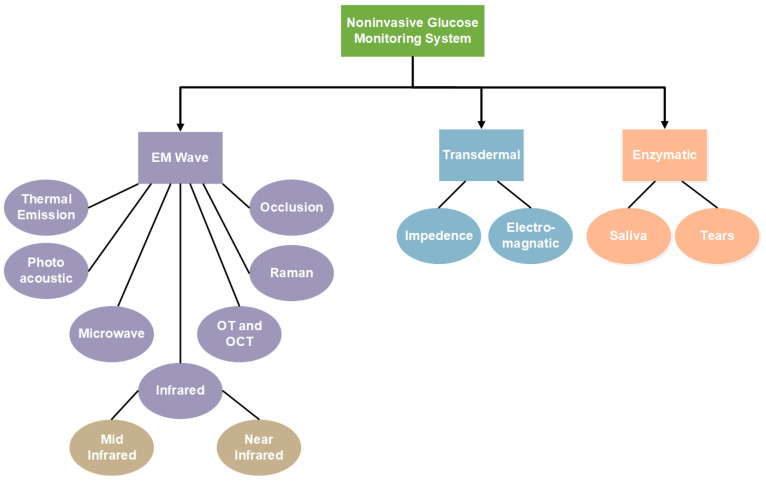
Noninvasive glucose monitoring system.

Noninvasive methods studied in recent years for glucose estimation can be grouped according to their technologies. Mainly, they fall into the category of electromagnetic (EM) wave sensing, transdermal, and enzymatic [11,12,13,14]. EM sensing comprehensively defines all the work related to the non-ionizing EM radiation, which includes ultraviolet (UV), infrared, microwaves, and the visible light spectrum. It includes Mid-Infrared (MIR), Near-Infrared (NIR), Microwave (MW), Thermal Emission (TE), Photoacoustic, Raman, and Occlusion spectroscopy. Optical Polarimetry (OP) and Optical Coherence Tomography (OCP) also fall into this category. Impedance spectroscopy and electromagnetic sensing are examples of transdermal technology, as they involve sensors being placed on the epidermis layer of human skin tissue. Enzymatic technology comprises noninvasive biological fluids such as saliva and tears for glucose measurements. Figure 1 shows the distribution of noninvasive technologies. Each of these technologies along with its advantages and disadvantages will be discussed in the following subsections.

### 2.1. Infrared (IR) Spectroscopy

**Figure 2:**
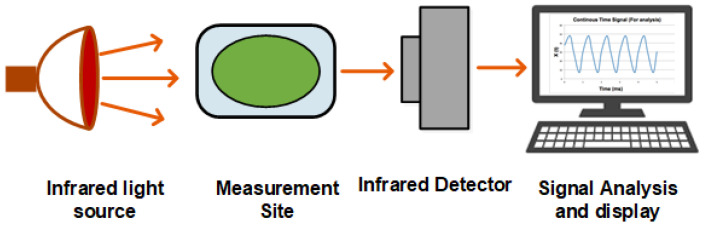
Infrared spectroscopy.

Infrared spectroscopy, or vibrational spectroscopy, is the measurement of how infrared radiations interact with matter. The absorption, emission, and reflection of IR waves are measured and studied to identify functional groups or chemical substances present in matter. The infrared region of the electromagnetic spectrum is usually divided into three sub-regions, the near-, mid-, and far-infrared, which are named by their relation to the visible spectrum. Figure 2 shows a general diagram of IR spectroscopy. For noninvasive glucose monitoring, mid-and near-infrared regions are more studied, as the penetration reduces with an increase in wavenumber. Both are discussed in the following subsections.

#### 2.1.1. Mid-Infrared (MIR) Spectroscopy

Mid-Infrared waves lie in the region of 2500–25 µm of electromagnetic waves. They use the reflection principle to detect glucose concentration in interstitial fluid (ISF) [15]. MIR has sharp response peaks for glucose detection and has low scattering. The main constraint of MIR is that can only be used in reflectance mode as it has poor penetration in human tissue and cannot determine glucose concentration present in blood vessels. The water content and human tissue affects the reflected light resulting in poor glucose correlation with ISF [16].

#### 2.1.2. Near Infrared (NIR) Spectroscopy

Near-Infrared spectroscopy is an optical method in which scattered, transmitted, or reflected light from the illuminated surface is studied. NIR waves lie in the EM bandwidth of 700–2500 nm. NIR spectroscopy finds its application in numerous fields like medicine, pharmaceutics, food analysis, quality control of chemical products, material sciences, astronomy, and agriculture [17,18,19,20,21,22,23]. It has been investigated for glucose estimation for the last few decades. NIR waves have deeper penetration compared to MIR, so they can easily reach the dermis layer of skin and interact with blood components. Thus, NIR spectrometry can be utilized to estimate glucose levels in the blood. This technology is low-cost and simple but suffers from scattering, and the lower bandwidth has a poor correlation with glucose in the blood. Another approach is to acquire PPG signals using NIR waves of specific bandwidths for blood glucose estimation [24,25]. PPG is an optical technique which detects volumetric changes in blood circulation. The PPG voltage signals are proportional to the quantity of blood flowing through the blood vessels. The changes in blood flow are seen as a waveform. The features obtained from these PPG signals are incorporated into machine learning algorithms to predict BGL. This technique has shown a better correlation with blood glucose. Section 3 will discuss NIR spectroscopy and its application in detail [26,27,28,29,30,31,32,33,34,35,36,37,38,39,40,41,42,43,44,45,46,47,48,49,50].

### 2.2. Raman Spectroscopy

Raman spectroscopy is based on measuring the scattering of incident monochromatic (laser) light due to the vibrational and rotational motion of particles under study. The change in light wavelength due to scattering (Raman shift) is measured to identify glucose molecules in which vibration modes are linked with carbon, hydrogen, and oxygen bonds [51,52]. Raman spectroscopy has a sharper spectrum compared with other infrared waves. It is less sensitive to water, ambient light, and temperature changes. The instability of the laser in wavelength and intensity is its major limitation. The intensity of the laser needs to be less to keep it harmless for the human body; therefore, it has a low signal-to-noise ratio (SNR).

### 2.3. Thermal Emission Spectroscopy (TES)

**Figure 3:**
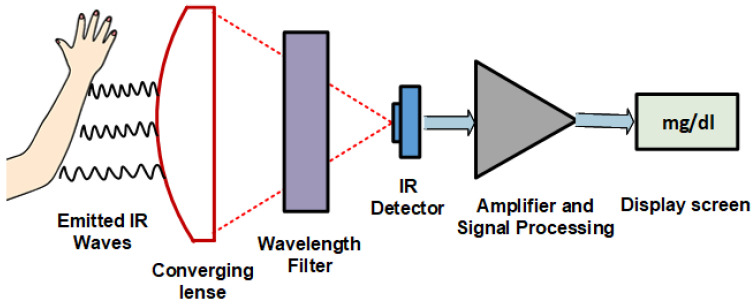
Thermal Emission Spectroscopy.

Thermal Emission Spectroscopy uses the heat radiation principle of the human body in the far-infrared region (8 µm–14 µm). When the body radiates heat, some of it is absorbed by different tissue and molecules, including glucose. The wavelength absorbing most is around 9.4 µm. Buchert [53] suggested that analysis of radiation can provide information on glucose concentration in blood. Figure 3 shows the setup of Thermal Emission Spectroscopy. Although TES is least sensitive toward scattering compared to other infrared waves, it has several limitations. The radiation intensity also depends upon temperature and measurement site thickness. It has strong water absorption, making accurate and sudden changes in glucose detection difficult.

### 2.4. Microwave Spectroscopy (MWS)

**Figure 4:**
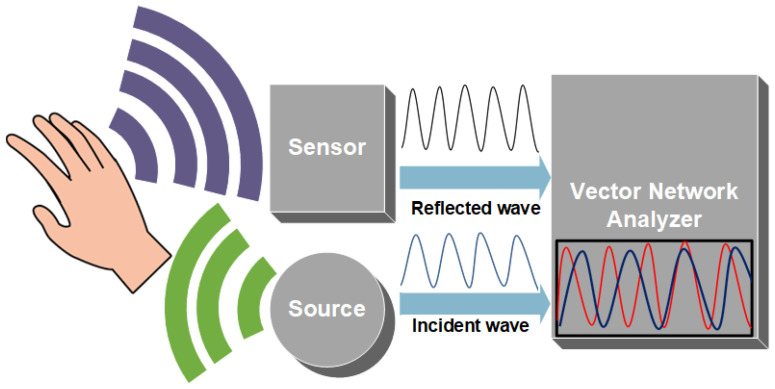
Microwave Spectroscopy working principle.

Microwaves range from 1 mm to 1 m in the EM wave spectrum. They are widely used in the fields of detection, communication, and medicine. As they can easily penetrate media with a millimeter of thickness, they can penetrate deep in skin tissue, reaching blood vessels in the dermis layer. The reflection, absorption, and transmission theory of microwaves through skin tissues can correlate to the changes in dielectric property, relative permittivity, and conductivity with fluctuating glucose concentrations [54,55,56]. Hence, implying these waves can be used to estimate BGL. Figure 4 shows the principle of MWS. The sensor is connected to a vector network analyzer (VNA) which detects changes in amplitude and phase corresponding to changes in permittivity of the sample, as shown in Figure 4. MWS is sensitive to a small glucose concentration; it can be easily designed and is low cost. Unfortunately, it has poor selectivity, as blood components affect the measurement parameters such as the dielectric constant. MWS is also sensitive to physiological parameters like breathing, sweating, and physical activities.

### 2.5. Metabolic Heat Conformation (MHC)

Metabolic Heat Conformation technology estimates blood glucose by measuring various physiological parameters using multi-wavelength spectroscopy methods along with humidity and temperature sensors [57,58]. The theory behind this technique is that the amount of glucose and oxygen levels present in the body correlate to the amount of heat produced by the metabolic oxidation of glucose in human cells. The heat emitted from the body as radiation, evaporation, and convention is measured via sensors and spectroscopies. Statistical analysis of the data is performed for glucose estimation. Although the physiological parameter is well-measured in this method, it has less accuracy, as it is susceptible to sweating and environmental conditions such as humidity and temperature variations.

### 2.6. Photoacoustic Spectroscopy (PAS)

Photoacoustic Spectroscopy exploits the photoacoustic method for glucose estimation. The theory states that if an energy source radiates on the skin surface, it causes thermal expansion at the illuminated site. Due to thermal expansion, acoustic or ultrasound waves are generated and can be detected by pressure sensors. The peak-to-peak variation of the detected signal can be correlated to the glucose level in the blood [59,60,61]. This technique is simple and is resistant to water absorption. For excitation sources, a wide range of laser pulses can be utilized, ranging from UV to NIR waves. However, this method is vulnerable to temperature, pressure, and environmental changes. It has a low signal-to-noise ratio and the instrumentation is expensive.

### 2.7. Occlusion Spectroscopy (OP)

Occlusion spectroscopy is a technique in which scattered light is measured from the pressurized tissue site. The blood flow is restricted for a few seconds at the site by applying pressure. The dynamic changes in blood flow increase the intensity of scattered light. The scattered light is measured to estimate blood glucose concentration [62]. Like other light sources, glucose estimation using OP is affected by physiological factors and ambient light sources.

### 2.8. Optical Polarimetry (OP)

Optical Polarimetry uses the concept of chiral molecules of glucose to estimate its concentration. Chiral molecules can rotate the polarization plane of the incident light beam at a particular angle. The amount of rotation is dependent upon the optical path length, temperature, wavelength of the incident beam, and concentration of an analyte. OP is unsuitable for skin measurements due to the high scattering of light and other physiological parameters. An aqueous humor of the eye is a suitable analyte for glucose estimation using OP [63,64]. This technique has high resolution and can measure small changes in aqueous glucose concentration but is sensitive to temperature changes and eye motion. The interferences from other optically active compounds in the eye result in poor specificity of his technique.

### 2.9. Optical Coherence Tomography (OCT)

Optical Coherence Tomography is an optical imagining technique that can give high-quality 2D images. The signal acquisition method is based on detecting interferometric signals. A low coherence light source is illuminated on the sample placed in an interferometer. The backscattered light from sample tissue and a reference mirror (inside the interferometer) forms an interferometric signal and is detected by a photodetector. With an increase in glucose concentration present in interstitial fluids, the refractive index also increases. The increase in refractive index decreases the scattering coefficient of illuminated light. Hence, measuring the scattering coefficient indirectly gives the glucose concentration present in the sample [65,66]. This technology has the advantage of good SNR, depth of penetration, and high resolution, but it suffers from tissue inhomogeneity, physiological interferences, and individual motion, resulting in poor selectivity for glucose estimation.

### 2.10. Bio-Impedance Spectroscopy

Bio-impedance measures the changes in permittivity and conductivity (impedance) through human tissue. The resistance to the flow of electric current through plasma fluid can be correlated to glucose molecules [67,68]. This is a relatively simple and easy-to-implement technique, but the error in measurements increases while sweating. This technique is also sensitive to temperature variation and other physiological conditions.

### 2.11. Electromagnetic Sensing

Electromagnetic sensing exploits the dielectric properties of blood to estimate glucose concentration. The fluctuation of voltage or current produced due to electromagnetic coupling of inductors indicates the varying concentration of blood glucose molecules [69,70]. Electromagnetic sensing is specific to the analyte and minimizes the interferences from surroundings, but it is highly sensitive to temperature changes.

### 2.12. Noninvasive Enzymatic Technology

Noninvasive enzymatic technology includes a technique that involves blood glucose using human fluids such as tears, saliva, and sweat. Ocular technology also falls in this category. It utilizes specially designed contact lenses that determine the glucose present in tears [71,72,73,74]. The saliva in the mouth has also been studied to detect the presence of glucose [75,76]. The main obstacle in noninvasive enzymatic technology is that these fluids do not necessarily depict glucose values of blood, and, hence, can give a false BGL estimation.

## 3. NIR Spectrometry

NIR spectrometry applicability has evolved in recent decades owing to significant improvements in spectral analysis and instrumentation. NIR spectrometry finds its application in various fields. In food analysis, the NIR technique is utilized to determine the chemical compositions and physical properties of bioactive compounds, commonly protein, fat, dry matter, and moisture content [17]. In agriculture, NIR spectroscopy is broadly applied for quality control purposes. Edible items include rice, fruits, vegetables, spices, oilseeds, dairy products, grains, tea, coffee, meat, fodder, and other related products. The reason for its extensive use is that it is non-reactive, accurate, low-cost, and easily deployed [18]. NIR spectrometry also finds its application in remote sensing and material sciences.

NIR spectroscopic imaging is employed to study the atmosphere, plants, and soil. NIR spectroscopy in material sciences is used in measuring the film thickness of microscopic samples and analyzing optical characteristics of nanoparticles and optical coatings. In the medical field, NIR spectrometry is used in medical imaging and physiological diagnostics [19]. It is now an established name for the analysis of body fluids and blood, for example, in ergonomics, sports training equipment, pulse oximetry, functional neuroimaging, medicine, neonatal research, brain-computer interface, urology, and neurology. NIR spectrometry still has more potential and is an active research area field [20].

Near-Infrared spectroscopy is a vibrational spectroscopy technique like Infrared and Raman spectroscopy. The interaction of molecules in the sample with electromagnetic waves stimulates the internal degree of freedom (DOFs) of molecules, i.e., bonds of the interacted molecules vibrate at different frequencies depending on the type and energy of the bonds. This determines the shape of the spectra of the sample. NIR can be divided into three regions according to its bandwidths: the first overtone band (1400–2000 nm), the second overtone band (750–1400 nm), and the combinational band (2000–2500 nm). NIR has a maximum penetration depth in the skin, compared to other infrared waves. Also, NIR is minimally absorbed by water and hemoglobin; hence, spectral measurements can be easily collected from skin or body surface.

The NIR waves are partially scattered or absorbed as they penetrate through skin tissue. As previously mentioned, the scattering and absorption are related to the molecular vibrations of chemical bonds of molecules present in the medium. This phenomenon can be utilized in measuring the concentration of biological functional groups such as C–H, N–H, C–O, and O–H present in the blood. Glucose molecules contain C–H and C–O bonds, so the absorption and reflectance of NIR waves passing through the skin can be developed to detect the concentration of glucose in the blood. The NIR absorption for isomers of glucose, such as fructose, lactose, and galactose, has absorption peaks at different wavelengths, ranging in first overtones and combinational bands of NIR spectra [21,22,23]. These wavelengths do not coincide with the wavelength (940 nm) at which glucose absorption is being detected. Hence, detection of glucose is not much affected by the presence of these isomers.

Many works in the literature show a detailed study of variations in NIR bandwidths and characteristic spectra due to changes in the concentration of glucose solution [24,25,26,27,28,29,30,31,32]. The study in [33] experiments with both shorter regions of NIR (700–1300 nm) and longer regions of NIR (1300–1700 nm) for glucose estimation. It concludes that despite higher regions of NIR bandwidth showing more prominent absorption by glucose, it is unable to penetrate through finger tissues due to considerable scattering in higher regions of the NIR spectrum. Hence, long NIR wavelengths cannot be used for the transmission method for noninvasive glucose measurements. For this reason, short NIR regions are more focused and studied for glucose estimation in blood. The next section discusses the recent studies of NIR for blood glucose estimation.

## 4. Blood Glucose Prediction Using NIR Techniques

Recent works in the literature show high accuracy in blood glucose prediction using NIR-based systems. They have been designed in reflectance and transmission modes. NIR spectroscopy, here, has been divided into two main subcategories: NIR spectrometry analysis and NIR PPG signal analysis. In NIR spectrometry analysis, logged voltage values after absorption and reflectance are measured, while in NIR PPG signal analysis, PPG signals are acquired using NIR LEDs. The following subsections will discuss them in detail.

### 4.1. NIR Spectrometry Analysis

Many experiments and studies verify the proof of concept to relate varying NIR intensities with BGL. They are briefly discussed. Yadav et al. [26] developed an NIR-based system for blood glucose measurements. They first develop a prototype for in vitro glucose measurements, using a 940 nm NIR LED. An increase in glucose concentration present in the solution showed a decrease in output voltages of the sensor. Later, they designed a sensor patch with an LED and photodiode. The sensor was placed on the forearm and took voltage measurements of the diffused reflectance spectra. Before and after a meal, spectra were analyzed. After the meal intake, a decrease in voltage level was observed, showing a correlation between voltage and glucose concentration. Before and after the meal, BGL was determined with a finger prick commercial glucometer, showing an increase in blood glucose concentration. Similar experiments were carried out by Buda [27] and Hotmartua [28].

These works on NIR-based systems confirmed the proof of the concept of a strong correlation between NIR absorption/attenuation with varying glucose concentrations. The work carried out consisted of mostly two phases. In first phase, simple in vitro lab experiments were performed to determine effect of different concentrated glucose solutions on NIR transmission. In the second phase, human subjects were involved and NIR voltage measurements were recorded for different times of day or before or after meal intake. The voltage variations were correlated linearly with glucose concentration.

**Figure 5:**
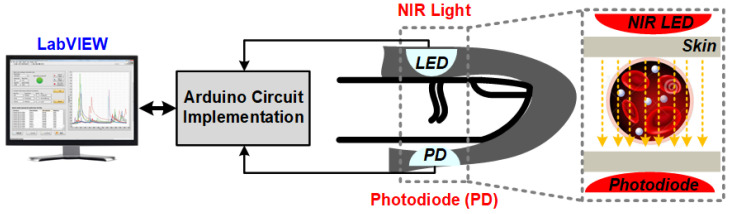
A prototype for the NIR transmission spectroscopy using a 940 nm wavelength for a noninvasive glucose monitoring system.

Studies carried out by Haxha [29], as shown in Figure 5, and Lee [30] confirm a strong correlation between NIR attenuation and glucose concentration. Jain [31] proposes a dual (940 nm and 1300 nm) short-wavelength NIR-wave-based detection system working on absorption and reflectance spectroscopy. Huber’s method-based regression model is utilized to improve the post-processing regression model. The proposed model develops a relationship between output voltages from the sensor and references blood glucose concentration (BGC) The model is developed using 25 subjects from the age group of 18–70 years. The reference blood glucose is measured using the finger-prick method (SD-check GOLD one-touch glucometer). The proposed system is validated on 200 individual subjects. System performance is evaluated using multiple metrics. The coefficient of determination (R^2^) value obtained is 0.9084; mARD is 5.18%. MAD is 6.25 mg/dL and RMSE is calculated to be 9.24 mg/dL. The model developed in this study has a limited number of training datasets. The study can be improved if the training dataset included more subjects with a wider range of blood glucose values.

Dai et al. [32] studied blood glucose detection using a 1550 nm NIR absorbance-based prediction model. The prediction model uses particle swarm optimization (PSO) and the two artificial neural networks (ANNs) model. This model considers a nonlinear relationship between light absorbance and blood glucose levels. Two ANNs serve as the basic structure of the overall model, and the weight coefficients of the two ANNs are improved by PSO. The Clarke error grid analysis of the proposed method shows that 64% of the predicted glucose values lie in region A; 29% of the predicted glucose value lies in region B; and 7% of predicted values lie in between regions A and D. The major shortcoming of this work is that the experimental datasets are very small. It only contains three average values from six healthy subjects. So, the accuracy of the proposed model needs to be verified over large datasets including diabetic patients.

Joshi et al. in [33] propose a wearable non-invasive consumer device that can be used by consumers for accurate continuous blood glucose monitoring. This device uses short NIR (940 nm and 1300 nm) wavelengths with absorbance and reflectance spectroscopy. It is integrated with the Internet-of-Medical-Things (IoMT) for smart healthcare. The deep neural network (DNN) regression model predicts glucose values. The performance of the device is validated with serum and capillary blood glucose. Data are collected from pre-diabetic, diabetic, and healthy patients with ages ranging from 17 to 80 years. The authors observe that the average error (AvgE) and mARD for serum glucose represents better results of calibration and validation compared to capillary glucose. The estimated samples of serum glucose values are observed at 100% in Clarke grid zone A. The blood glucose prediction of the device has a range of 80–420 mg/dL. The performance of the device is given by Average Error (AvgE) and Mean Absolute Relative Difference (mARD). For capillary blood glucose, AvgE and mARD are calculated as 6.09% and 6.07%, respectively, whereas for serum glucose, AvgE and mARD are estimated as 4.88% and 4.86%, respectively.

### 4.2. NIR PPG Signal Analysis with Machine Learning

**Figure 6:**
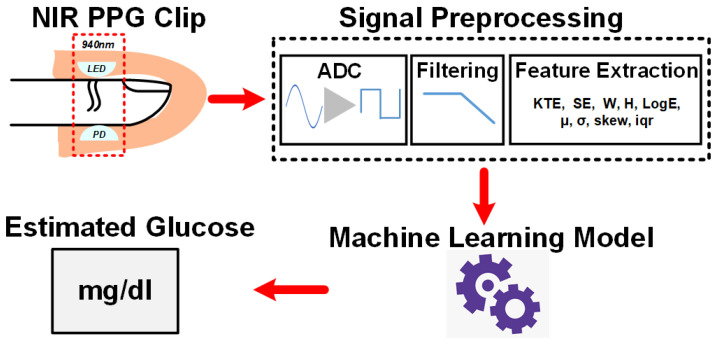
Block diagram of the NIR PPG signal glucose sensing platform with machine learning.

**Figure 7:**
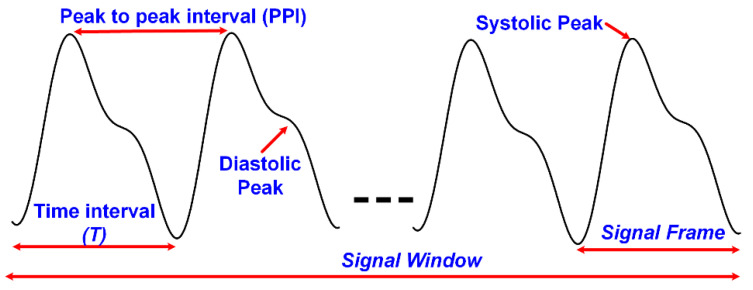
PPG waveform and its basic features.

PPG signals are unique, as they contain important information. The fingertip PPG waveform reflects the blood flow in blood vessels pumping from the heart to the fingertip. Its well-defined peaks represent the systole and diastole of heart rate [34]. The PPG pulse can be divided into two portions of the cardiac cycle; the rising part of the pulse relates to systole, and the falling part of the pulse represents the diastole. PPG signals have become of much interest, as they are noninvasive, low-cost, and suitable diagnostic tools. They are already being used in the measurement of oxygen saturation, blood pressure, and heart rate (BPM) [35,36,37]. A full understanding of the different features is still lacking. Over the last decade, researchers have been studying PPG signals for noninvasive glucose monitoring. This section focuses on BGL estimation by machine learning algorithms using features extracted from NIR PPG signals. A generic block diagram of this glucose sensing platform is shown in Figure 6. The PPG signal waveform with some of its basic features is shown in Figure 7. The following paragraphs will discuss some of the related research works.

Monte-Moreno’s study [38] is one of the initial works which investigates noninvasive BGL estimation using PPG signals with machine learning algorithms. This system also measures blood pressure and heart rate using the same recorded PPG signals, and 410 individual data are recorded. The designed module also mitigates motion artifacts and rejects noisy recorded signals. After signal processing, main features are extracted, and a feature vector of fixed length is formed. This feature vector is input into the machine learning module. The PPG signals are recorded for a 1 min duration. Each signal is segmented into overlapping frames of length 5 s. Both segmented and unsegmented signals are input to the feature extraction unit. The statistical features extracted from input signals are mean, variance, skewness, and interquartile range (IQR) of Kaiser Teager Energy (KTE), heart rate (HR), spectral entropy (SE), IQR, and variance of Log Energy (LogE) profiles. Autoregressive (AR) coefficients of order 5 from the PPG waveform, KTE, and LogE are also computed. Other non-statistical features include oxygen saturation range (OSR), age (AG), weight (WGT), and body mass index (BMI). The feature vector (XF) is formed by concatenating all the features. This feature vector is input to the machine learning module. In total, a 33-dimensional feature vector is formed from 20 distinct features given by:

(1) $$X_{F}={\left[\begin{array}{c} {\mathrm{KTE}}_{\mathrm{AR}},{\mathrm{KTE}}^{\mu },{\mathrm{KTE}}^{\sigma },{\mathrm{KTE}}^{\mathrm{iqr}},{\mathrm{KTE}}^{\mathrm{skew}},{\mathrm{SE}}^{\mu },{\mathrm{SE}}^{\sigma },{\mathrm{SE}}^{\mathrm{iqr}},{\mathrm{SE}}^{\mathrm{skew}},\\ {\mathrm{HR}}^{\mu },{\mathrm{HR}}^{\sigma },{\mathrm{HR}}^{\mathrm{iqr}},{\mathrm{HR}}^{\mathrm{skew}},{\mathrm{LogE}}^{\sigma },{\mathrm{LogE}}^{\mathrm{iqr}},{\mathrm{LogE}}_{\mathrm{AR}},{\mathrm{AR}}_{\mathrm{PPG}},\mathrm{OSR},\mathrm{WGT},\mathrm{BMI} \end{array}\right]}^{T}$$

Performance factors of different machine algorithms compared are Linear Regression (LR), neural network (NN), Support Vector Machines (SVM), and Random Forest (RF). In the simulation, Random Forest performs better than the rest, and, hence, is selected for the testing dataset. The coefficient of determination of test subjects, with the Random Forest trained model, has an R^2^ of 0.88. The overall system performance has an R^2^ of 0.9.

Rachim [39] proposed a cost-effective and wearable blood glucose detecting sensor. It has a small data acquisition time window and can be used as a continuous blood glucose monitoring (CGM) system. The proposed system is worn around the wrist and uses visible and NIR spectroscopy. Two NIRs and two visible LEDs are chosen for this purpose. The reflected optical signal is measured. A total of 12 volunteers are studied and given carbohydrate-rich meals. The optical signal is recorded as PPG signals. The PPG signals are then processed for feature extraction. The 24 extracted features from the PPG are input to the prediction model. Partial Least Squares (PLS) are used as a calibration algorithm to find a relationship between features and glucose concentration. A 10-fold cross-validation procedure is used to validate the model. The proposed system shows an average correlation coefficient of 0.86 with a standard prediction error of 6.16 mg/dL.

Ramashyamam et al. [40] designed a system for non-invasive blood glucose estimation using PPG NIR spectroscopy. The analog frontend comprises a photodetector and three NIR LEDs of wavelengths 1070 nm, 950 nm, and 935 nm, and with an optodes pair. The recorded PPG signals are processed, and double regression analysis is performed with the artificial neural network. The proposed system is implemented on FPGA. To improve accuracy of neuron weights in ANN, an inverse delayed (ID) function was used. A significant reduction of error was observed in predicted glucose values using the Inverse Delayed (ID) function ANN neuron model, as compared to the conventional ANN neuron model. The mean square error was reduced from 5.84 mg/dL to 1.02 mg/dL. The neural networks were trained and tested using MATLAB software. The analysis showed that increasing the number of hidden neurons increased the accuracy of glucose prediction. Hence, 15 hidden neuron layers were used in the model. The system performance has been verified on 50 patients. The authors claim an accuracy of 94% for the proposed system.

The study in [39,40] needs to develop the proposed prototypes to an optimal device to verify the sensor reproducibility. It also needs to test the device on more human subjects with a diversity in the region.

Chu et al. [41] studied a PPG-based noninvasive blood glucose prediction with a large number of subjects (2538) and observed a reduced accuracy of prediction. They suggested that the reason for reduced accuracy is the physiological diversity of subjects, one of them being medication. So, they divided their dataset into two cohorts with and without medication and predicted glucose values by deep learning. They found out that without medication, the cohort has a 30% higher accuracy than for the medication cohort. They also included a quarterly measured HbA1c with both groups and observed an increase in prediction accuracy of 10%. The coefficient of determination value for the un-medicated cohort with a quarterly measured HbA1c is 0.71 with a RMSE of 12.4 mg/dL.

Habbu et al. [42] presented an NIR-based blood glucose estimation system. Apart from working with similar PPG signal features as those in [38], some new features were also explored for the estimation of BGL values. Data were collected from 611 individuals with blood glucose values ranging from 70 mg/dL to 450 mg/dL. The PPG signals were recorded for around 3 min. The extracted features were divided into two sets: a) time and frequency domain (TFD) features and b) Single Pulse Analysis (SPA). The time and frequency domain feature includes similar statistical features as in [38] for BGL estimation. The extracted features are the mean, variance, skewness, and IQR of spectral entropy (H); skewness and autoregressive coefficients of Kaiser Teager Energy (KTE); mean and skewness of heart rate (HR); and IQR and variance of Log Energy (LogE) profiles. Autoregressive (AR) models of order 5 from PPG waveform and LogE are similarly calculated. In addition to these features, the time-domain features, pulse transit time (PPint), peak-to-peak interval (PPI), and pulse amplitude (Pamp) are extracted. They are added to the feature vector. In total, a 35-dimensional feature vector is formed from 15 distinct features. The 15 distinct feature vectors (FV) given below are input into the NN training module.

(2) $$\mathrm{FV}={\left[\begin{array}{c} {\mathrm{KTE}}_{\mathrm{AR}},{\mathrm{KTE}}^{\mathrm{skew}},H^{\mu },H^{\sigma },H^{\mathrm{iqr}},H^{\mathrm{skew}},{\mathrm{HR}}^{\mu },{\mathrm{HR}}^{\mathrm{skew}},\\ {\mathrm{LogE}}^{\sigma },{\mathrm{LogE}}^{\mathrm{iqr}},{\mathrm{LogE}}_{\mathrm{AR}},{\mathrm{AR}}_{\mathrm{PPG}},\mathrm{PPT},\mathrm{Pamp},\mathrm{PPint} \end{array}\right]}^{T}$$

In Single Pulse Analysis, the pulses are separated within a given window, and features such as pulse energy, pulse transit time, pulse interval, pulse amplitude, pulse onset, and end time, etc., are calculated. After that, their statistical parameters such as mean, variance, IQR, and skewness (similar to work [38]) are also calculated. In total, a feature vector of 28 dimensions, originating from four basic feature-distinct features, is input into the training model:

(3) $$\mathrm{FVnew}={\left[{\mathrm{PFV}}_{n}^{\mu },{\mathrm{PFV}}_{n}^{\sigma },{\mathrm{PFV}}_{n}^{\mathrm{skew}},{\mathrm{PFV}}_{n}^{\mathrm{iqr}}\right]}^{T}$$

This feature vector is input into the NN model for training. The performance of both models is seen in the test dataset. The value of the coefficient of determination for time and frequency domain features (FV) is R^2^ = 0.84, while for SPA, it is R^2^ = 0.91, showing a better performance comparatively. The Clarke error grid analysis for BGL estimation for both cases was completed. Using the time and frequency domain feature set, the data distribution was 80.6% in class A and 17.4% in class B. For SPA, the data distribution was 83% in class A and 17% in class B, showing a better estimation.

Yadav et al. [43] developed a multi-sensor prototype for glucose estimation. They also worked with similar features with the addition of some physiological parameters. A close group study of 50 normal subjects was conducted. The first dataset was recorded in a fasting condition. The second dataset was recorded after each subject consumed 75 g of glucose in 300 mL of water. A total of 4 recordings of every individual were completed with 30 min intervals. Apart from PPG signal features (Kaiser Teager Energy (KTE), heart rate (HR), and entropy (H)), other parameters include skin temperature (temp), galvanic skin response (GSR), and person-specific information (age (AG), body mass index (BMI), and weight (Wt)). The 17 distinct feature vectors, Y^n^, are given in Equation (4).

(4) $$Y^{n}=\left[\begin{array}{c} {\mathrm{KTE}}_{n}^{\mu },{\mathrm{KTE}}_{n}^{\sigma },{\mathrm{KTE}}_{n}^{\mathrm{iqr}},{\mathrm{KTE}}_{n}^{\mathrm{skew}},H^{\sigma },H^{\mu },H^{\mathrm{iqr}},H^{\mathrm{skew}},{\mathrm{HR}}^{\mu }\\ {\mathrm{HR}}^{\sigma },{\mathrm{HR}}^{\mathrm{iqr}},{\mathrm{HR}}^{\mathrm{skew}},\mathrm{GSR},\mathrm{temp},\mathrm{AG},\mathrm{BMI},\mathrm{Wt} \end{array}\right]$$

Blood glucose levels were estimated using multiple linear regression (MLR) and ANN. For ANN, a Multilayer Perceptron (MLP) network was trained using the Levenberg–Marquardt (LM) algorithm. Using the MLR technique, the correlation coefficient, R^2^, calculated was 0.612. Using ANN, the correlation coefficient of test data significantly improved to 0.96.

Similar features sets are used in work [38,42,43]. The work needs to include a greater number of subjects, particularly diabetic patients. The feature vector formed contains many statistical and physiological parameters. This may adversely affect the results for a larger training and testing dataset, so a feature vector with an optimal number of features needs to be investigated. The proposed system also needs to be implemented on hardware and tested.

Hina et al. [44] presented a system working with a single wavelength of 940 nm for frequent glucose monitoring. The NIR PPG signals are recorded for over a minute and features are extracted. Different machine learning models were trained using extracted features and reference glucose values. The performances of all the trained models were evaluated on test data. The best performing model was selected for hardware implementation. The proposed system is implemented on a chip using a 180 nm CMOS process, with an area of 4.0 mm^2^. The designed SoC consumes 1.62 mW of power. The PPG readout architecture comprises a transimpedance amplifier of 1 MΩ gain and a switched capacitor low pass filter of 10 Hz. The input-referred current noise of the analog front-end is 7.3 pA/√Hz, and it allows a DC bias current rejection up to 20 µA. The implemented digital processor removes baseline drifts from the recorded PPG signal, extracts six features, and predicts BGL using Support Vector Regression (SVR) with a Fine Gaussian kernel (FGSVR). A piece-wise linear (PWL) approach is used for the exponential function FGSVR implementation on-chip. Using the proposed SoC, 200 healthy and diabetic subjects are tested. The system performance is evaluated with mARD and R square. It achieves an mARD of 7.62 and an R^2^ of 0.937. The authors [44,45,46] also use a similar feature set as [38] for glucose estimation. PPG signals of 1 min are recorded from diabetic and non-diabetic subjects. PPG signals are often corrupted with noises and motion artifacts. Before feature extraction, filtering is performed. Different filter performances are studied, and the moving average filter is selected for convenient hardware implementation. After removing baseline drifts from the signals, each signal is segmented into non-overlapping frames of a fixed length. From each signal, 14 distinct features are extracted. The mathematical operators calculated are Kaiser Teager Energy (KTE), LogE, and spectral entropy (SE). Like previous work, four statistical features, mean, variance, interquartile range, and skewness, of these mathematical operators are extracted. Autoregressive coefficients (AR) of the order three for PPG signals, KTE, and LogE are also calculated. Combining all the 14 distinct features, a 23-dimensional feature vector is formed. The feature vector (X_F_) is given by:

(5) $$X_{F}={\left[\begin{array}{c} {\mathrm{AR}}_{\mathrm{PPG}},{\mathrm{KTE}}_{µ},{\mathrm{KTE}}_{\sigma },{\mathrm{KTE}}_{\mathrm{iqr}},{\mathrm{KTE}}_{\mathrm{skew}},{\mathrm{AR}}_{\mathrm{KTE}},{\mathrm{SE}}_{µ},\\ {\mathrm{SE}}_{\sigma },{\mathrm{SE}}_{\mathrm{iqr}},{\mathrm{SE}}_{\mathrm{skew}},{\mathrm{LogE}}_{µ},{\mathrm{LogE}}_{\sigma },{\mathrm{LogE}}_{\mathrm{iqr}},{\mathrm{AR}}_{\mathrm{LogE}} \end{array}\right]}^{T}$$

In this work, the authors also determine the optimal number of features needed for the best glucose estimation. Using the Wrapper method, different combinations of features are studied to see how many features and which combination set performs better than others. The performance of different machine learning algorithms is also studied for the set of combinations. Table 1 shows the summary performance of five different combinations of feature sets for four machine learning algorithms. Support Vector Regression (SVR) with a Fine Gaussian kernel (FGSVR) with six features shows the best performance and has an optimized number of features. Hence, this training model is selected for BGL estimation.

Hina A. et al. [47] use a new set of features for glucose estimation. The PPG signal is recorded and fragmented similarly. From each PPG signal, six distinct features are extracted. These extracted features are peak-to-peak interval (PPI), Power Spectral Density (S_PD_), Detrended Fluctuation Analysis (S_F(W)_), and wavelet entropy (W_PPG_). Three statistical features are extracted from PPI, which are the standard deviation of peak-to-peak amplitudes, the standard deviation of peak-to-peak intervals (kurtosis), and the Mean Absolute Deviation (MAD) of peak-to-peak amplitudes (PPA). The input vector formed is given by:

(6) $$X_{F=}\left[{\mathrm{PPI}}_{\mathrm{std}},{\mathrm{PPI}}_{k},{\mathrm{PPI}}_{\mathrm{MAD}},S_{F\left(w\right)},S_{\mathrm{PD}},W_{\mathrm{PPG}}\right]$$

These features show a better performance with ensemble boosted trees, as seen in Table 2; hence, they are chosen for glucose estimation. The mARD shows improvement from the last work. It has a value of 5.83. The mathematical details of the features from [44,47] will be discussed in the next section, Section 4.3.

### 4.3. Mathematical Details of PPG Features

This section briefly describes the mathematics of the features extracted from PPG signals in thework [44,45,46,47].

(1) Kaiser Teager Energy feature (KTE)

Kaiser Teager Energy helps in determining the instant energy profiles of signals [77,78,79]. The operators are defined for both the continuous and discrete time domain. Energy functions can be written as a product of simpler functions using KTE operators. Signal analysis from KTE operators can indicate the quality of a signal. A high mean KTE refers to a clear signal, while a low mean KTE refers to a noisy signal. This, in turn, may help in deciding to keep or discard a signal. The KTE operator for a discrete signal x[n] is as follows:

(7) $$\mathrm{KTE}\left[n\right]=x^{2}\left[n\right]-x\left[n-1\right]x\left[n+1\right]$$

For PPG signal analysis, KTE features are calculated for a whole signal window and frame level signal as well. KTE for window level is given by Equation (8), while for frame level, it is given by Equation (9):

(8) $${\mathrm{KTE}}_{w}\left(k\right)=S_{w}^{2}\left(k\right)-S_{w}\left(k-1\right)\times S_{w}\left(k+1\right)$$

(9) $$\mathrm{KTE}\left(\tau ,n\right)=S_{f}^{2}\left(\tau ,n\right)-S_{f}\left(\tau +1,n\right)\times S_{f}\left(\tau -1,n\right)$$

where n = 1, 2, 3 … 10, and τ = 2, 3, 4 … L_frame_ − 1. The value of L_frame_ is 128 for the work in [38,42,45,46]. From Equation (9), the variance $\left({\mathrm{KTE}}_{\sigma }\right)$, mean $\left({\mathrm{KTE}}_{µ}\right)$, skewness [80] $\left({\mathrm{KTE}}_{\mathrm{skew}}\right)$, and the interquartile range $\left({\mathrm{KTE}}_{\mathrm{IQR}}\right)$ are determined.

(2) Logarithmic Energy feature (LogE)

Logarithmic Energy is a time-domain feature which measures energy of the signal. It is commonly computed from a full-band spectrum of the signal [81]. The LogE feature is computed from each frame of the signal and is given by:

(10) $$\mathrm{LogE}=\log\left(\sum_{\tau =1}^{L_{\mathrm{frame}}}S_{f}^{2}\left(\tau ,n\right)\right)$$

where n is the number of frames and τ is the index number of a sample in the frame. From the computed LogE, the statistical features such as interquartile range $({\mathrm{LogE}}_{\mathrm{iqr}}),$ mean $\left({\mathrm{LogE}}_{µ}\right)$, and variance $\left({\mathrm{LogE}}_{\sigma }\right)$, and are calculated.

(3) Autoregressive model of PPG signal

AR coefficients are commonly used for extraction of respiratory rate from PPG signals [82]. AR coefficients are calculated both at the window and frame level for different features. At the window level, AR coefficients of the PPG signal, KTE, are calculated. From the frame level, an AR coefficient of LogE is calculated. Three distinct feature vectors are formed, AR_PPG_, AR_KTE,_ and AR_LogE_. These are vector quantities where the dimension is one, plus the order of the AR model selected. The authors in [44] uses the AR model of order three. The AR models of the features $S_{w},{\;\mathrm{KTE}}_{w},$ and LogE are given as:

(11) $$S_{w}\left(n\right)=-\overset{p}{\underset{i=1}{\sum }}{\mathrm{AR}}_{\mathrm{PPG}}{}_{i}S_{w}\left(n-i\right)+e\left(n\right)$$

(12) $${\mathrm{KTE}}_{w}\left(n\right)=-\overset{p}{\underset{i=1}{\sum }}{\mathrm{AR}}_{\mathrm{KTE}}{}_{i}{\mathrm{KTE}}_{w}\left(n-i\right)+e\left(n\right)$$

(13) $$\mathrm{LogE}\left(n\right)=-\overset{p}{\underset{i=1}{\sum }}{\mathrm{AR}}_{\mathrm{LogE}}{}_{i}\mathrm{LogE}\left(n-i\right)+e\left(n\right)$$

where e(n) is the prediction error in the estimated model, p is the order of the AR model, and ${\mathrm{AR}}_{\mathrm{PPG}}{}_{i},{\;\mathrm{AR}}_{\mathrm{KTE}}{}_{i},{\;\mathrm{and}\;\mathrm{AR}}_{\mathrm{LogE}}{}_{i}$ are the coefficients of the model.

(4) Spectral entropy feature (SE)

Spectral entropy approximates the spectral power distribution of a given signal. It determines the damping of pulses, spectral shape, and the harmonic components of a signal. Using spectral entropy, a signal can be examined to be noisy or missing some information. Spectral entropy has also been used in speech signal detection in previous literature work [83,84,85]. This concept has been utilized in literature work to correlate the range of SE values with glucose levels in the blood. The Equations (14)–(16) compute SE. The features extracted from SE are variance $\left({\mathrm{SE}}_{\sigma }\right)$, mean $\left({\mathrm{SE}}_{µ}\right)$, skewness $\left({\mathrm{SE}}_{\mathrm{skew}}\right)$, and the interquartile range $\left({\mathrm{SE}}_{\mathrm{iqr}}\right)$.

(14) $$X_{n}\left[k\right]=\mathrm{FFT}(S_{f}\left(\tau ,n\right)$$

(15) $$P_{X}^{n}\left[k\right]=\frac{{\left|X_{n}\left[k\right]\right|}^{2}}{\sum_{j=1}^{L_{\mathrm{frame}}}{\left|X_{n}\left[j\right]\right|}^{2}},k=1,2\ldots L_{\mathrm{frame}}$$

(16) $$\mathrm{SE}=\overset{L_{\mathrm{frame}}}{\underset{k=1}{\sum }}P_{X}^{n}\left[k\right]\mathrm{Log}\left(P_{X}^{n}\left[k\right]\right)$$

The Fourier transform of signal S_f_ (τ, n) is the vector $X_{n}\left[k\right]$ of length k = 128, given in Equation (14). Equation (16) calculates SE from the normalized term,${\;P}_{X}^{n}\left[k\right]$, given in Equation (15).

(5) Peak-to-peak interval (PPI)

Peak-to-peak interval (PPI), as understood by the name itself, is the distance between two peaks of a signal. In PPG signal analysis, it reflects the cardiac cycle of the human body. It is a time-domain analysis of the signal and is used to determine heart rate. The three statistical features that are derived from PPI are the standard deviation of peak-to-peak intervals (kurtosis) in Equation (17), the Mean Absolute Deviation (MAD) of peak-to-peak amplitudes (PPA) in Equation (18), and the standard deviation of peak-to-peak amplitudes given in Equation (19).

(17) $$\mathrm{kurtosis}\left(k\right)=\frac{\frac{1}{N}\sum_{i=1}^{N}{\left(S_{i}-\bar{S}\right)}^{4}}{std^{4}}$$

(18) $${\mathrm{PPA}}_{\mathrm{MAD}}=\frac{\sum_{i=1}^{Np}\left|A_{i}-\hat{A}\right|}{Np}$$

(19) $$td=\sqrt{\frac{1}{N}\overset{N}{\underset{i=1}{\sum }}{\left(S_{i}-\bar{S}\right)}^{2}}$$

where *N* is the total number of samples in the signal, $\bar{S}$ is the mean value of the signal, *A* is peak-to-peak amplitude, and *Np* is the number of peak-to-peak intervals in 1 min of recorded PPG signals.

(6) Detrended Fluctuation Analysis (DFA)

Detrended Fluctuation Analysis is a time-domain analysis of a signal. DFA is a statistical method to compute correlations that have a long range in a time series [86]. An integrated time series can be calculated by the following formula:

(20) $$y\left(k\right)=\overset{N}{\underset{i=1}{\sum }}\left(S\left(i\right)-\bar{S}\right)$$

where *S*(*i*) is the length of the signal. The signal is divided into segments of width, w. Any integer value is selected for width after computational analysis. In the work of [47], the length *w* = 10 is chosen. The average fluctuation of the signal, $S_{F\left(w\right)}$, is calculated as follows:

(21) $$S_{F\left(w\right)}=\sqrt{\frac{1}{N}\overset{N}{\underset{k=1}{\sum }}(y\left(k\right)-y_{n}\left(k\right){)}^{2}}$$

(7) Power Spectral Density (PSD)

The Power Spectral Density is a frequency domain feature. The distribution of power signal over frequency is revealed by PSD analysis. It is defined as:

(22) $$S_{PD}=\underset{T\to \infty }{\lim}E\left[{\left|{\hat{S}}_{T}\left(\omega \right)\right|}^{2}\right]$$

where ${\hat{S}}_{T}\left(\omega \right)$ is the Fourier transform of the incoming signal, *S*(*i*).

(8) Wavelet entropy (WE)

Using wavelet entropy [87], three statistical features are computed. The transient features of the PPG signals are analyzed by wavelet entropy. This analysis combines entropy and wavelet decomposition to estimate the amount of disturbance in the signal, with high frequency-time resolution. The wavelet functions provide more insight into the extracted features, as they are localized in the time and frequency domain and have multi-resolution analysis. The wavelet transform defines the incoming PPG signal as a coefficient and represents time-domain features at different resolutions. From these wavelets transforms, wavelet entropy is calculated:

(23) $$W_{PPG}=-\underset{j}{\sum }P_{j}\ln\left(P_{j}\right)$$

(24) $$P_{j}=\frac{E_{j}}{E_{tot}};\;E_{j}=\sum {\left|C_{j}\left(i\right)\right|}^{2};\;E_{tot}=\sum E_{j}$$

where j = 1, 2, 3 … 4 denotes the layers of decomposition; i = 1, 2, 3, 4 … 1280 are number of samples, $P_{j}$ is the probability of layers, $E_{j}$ is the energy at each respective layer, and $E_{tot}$ is the total energy of layers.

### 4.4. BGL Estimation Analysis and Comparision Table

Table 3 shows the performance of the aforementioned state-of-the-artwork for glucose estimations. The table compares the extracted number of features used in BGL estimation, the implemented machine learning algorithms, and the coefficient of determination R^2^ for the test datasets.

**Figure 8:**
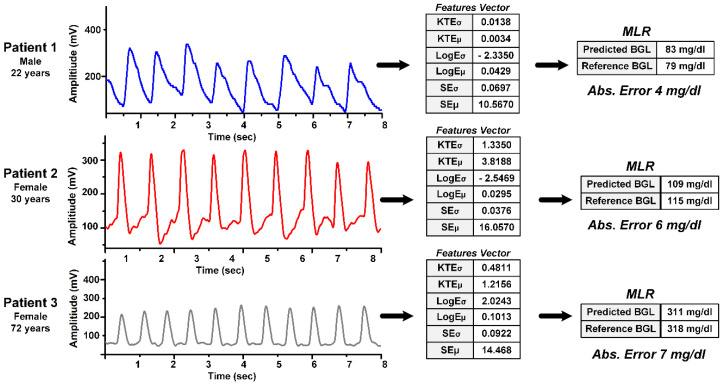
Measured PPG signals with BGL estimation for 3 different subjects having reference a BGL of 79, 115, and 318 mg/dL, respectively. Reprinted with permission from [44].

The PPG signals from three different subjects are shown in Figure 8 and taken from [44]. The figure shows the measured PPG signals from three random patients, extracted feature vectors, and estimated BGL. The predicted glucose by the proposed system is quite close to reference values as seen in the figure.

**Figure 9:**
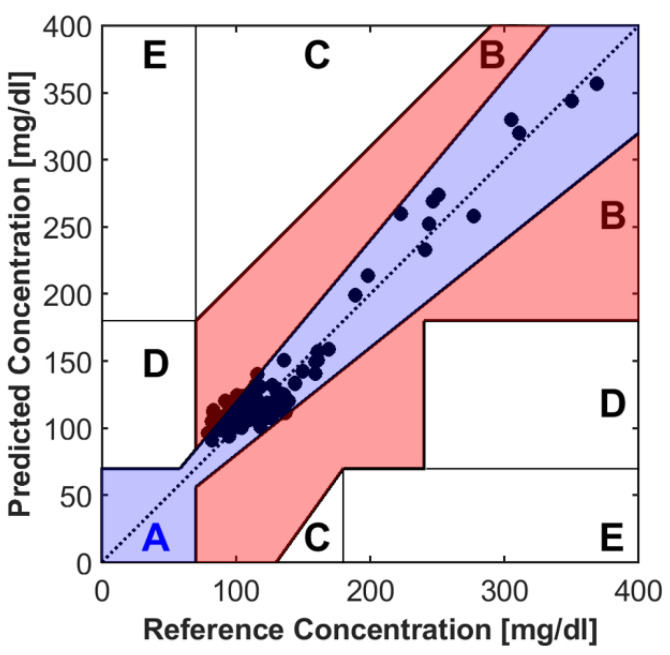
The Clarke error grid analysis of estimated and reference BGL. Reprinted with permission from [44].

The Clarke grid error analysis (EGA) is the golden standard for analyzing the estimation performances of proposed blood glucose monitoring systems [88]. The Clarke Grid is divided into five main regions or zones. Region or zone A is for the estimated glucose values within 20% of the reference value, and region or zone B is for the estimated glucose values with more than 20% of the reference value and does not cause wrong treatment. Regions C, D, and E are error regions or zones, and are considered clinically unacceptable. The Clarke grid analysis for the proposed system in [44] is seen in Figure 9. More than 95% of predicted glucose values lie in zone A, which is a clinically acceptable zone with least errors, the rest of the 5% of values lies in zone B. The performance of the Clarke grid indicates high accuracy of the proposed BGL monitoring system. It achieves a mARD of 7.62% and an RMSE of 11.20.

## 5. Discussion

So far, the experiments and research conducted using NIR technology focus more on data collection from adult volunteers. Adult volunteers include healthy subjects and diabetes patients having Type II diabetes. The studies need to expand the dataset to include a greater number of diabetic subjects suffering with hypoglycemia. These includes patients of type I diabetes. Apart from adding more subjects, diversity needs to be added in subjects too. The data collection should be expanded to different cities. Volunteers from different regions should be encouraged to participate. This will help in developing a generalized training models for machine learning methods. The reproducibility of a sensor is of equal importance. For checking the reproducibility of the sensor in work [45], the data have been collected from different places spanning months. The sensor has been used several times for data collection in university campuses, cafes, and homes (volunteers willing to collect samples from their homes). The diabetic patient data has been mostly collected from Lahore Diabetic Centre. The reproducibility can be further checked while collecting a diverse dataset. NIR PPG signals are prone to motion artifacts. More research work is needed to develop a robust device that can mitigate motion artifacts and not affect BGL estimations.

## 6. Conclusions

As diabetic patients are increasing day by day, there is a need to develop a noninvasive continuous glucose monitoring system for better diabetic management. Researchers are working to develop a device that is painless, cost-effective, and accurate. NIR spectrometry has great potential in noninvasive glucose monitoring. This review paper summarizes the recent works in noninvasive blood glucose monitoring with a focus on NIR technologies using PPG signals with machine learning. The mathematical details of the features extracted are discussed briefly. Work with similar features is compared. Although the works show promising results, most of them only show simulation results for the test data. More extensive research is required to develop an NIR-based noninvasive BGL system.

For acquiring PPG data, different NIR wavelengths or pulse oximeters are used in the literature. To have a more precise comparison, PPG data should be acquired using similar wavelengths. The number of features used for the training model also varies among the previous works. A deeper feature analysis is needed to determine and optimize the number of features that will be sufficient for glucose estimation. A much larger dataset acquisition is required for better training of ML models. Most of the literature’s work on NIR technologies is software-based. There is a need to develop and design cost-effective and low-power hardware that can process incoming, real-time data for glucose estimation. Only then can the noninvasive system performance be truly evaluated. Later, this system can be further developed into a wearable device for continuous glucose monitoring.

## Figures and Tables

**Table 1:** Different feature set performance with four machine learning algorithms.

	SVR-Fine Gaussian		SVR Quadratic		Linear Regression		En. Boosted Trees	
Combination of Features	mARD	RMSE	mARD	RMSE	mARD	RMSE	mARD	RMSE
AR_PPG_, KTE_σ_, KTE_µ_, KTE_iqr_, KTE_skew_, AR_KTE_, LogE_σ_, LogE_µ_, LogE_iqr_, AR_LogE_, SE_σ_, SE_µ_, SE_iqr_, SE_skew_ (14 Features)	8.36	11.29	18.27	25.21	22.57	33.85	18.27	25.21
KTE_σ_, KTE_µ_, KTE_iqr_, KTE_skew_, LogE_σ_, LogE_µ_, LogE_iqr_, SE_σ_, SE_µ_, SE_iqr_, SE_skew_ (11 Features)	10.16	12.31	15.01	46.00	14.66	26.00	15.24	21.64
KTE_σ_, KTE_µ_, KTE_iqr_, KTE_skew_, LogE_σ_, LogE_iqr_, SE_σ_, SE_µ_, SE_iqr_, SE_skew_ (10 Features)	13.66	21.93	22.09	44.41	16.19	29.94	16.18	23.00
KTE_σ_, KTE_µ_, KTE_iqr_, KTE_skew_, LogE_σ_, LogE_iqr_, SE_σ_, SE_µ_, SE_iqr_, SE_skew_ (8 Features)	12.17	21.6	22.05	50	19.19	25.86	16.05	23.21
KTE_σ_, KTE_µ_, LogE_σ_, LogE_μ_, SE_σ_, SE_µ_ (6 Features)	7.62	11.20	21.10	42.90	13.22	23.35	9.67	13.00

**Table 2:** Performance of different machine learning algorithms for the new feature set.

Machine Learning Algorithm	mARD	RMSE
Linear Regression	8.25	12.35
Fine Gaussian	7.36	11.20
Non-Linear Medium Gaussian	6.52	10.15
Ensemble Boosted Trees	5.83	8.65

Taken from NEWCAS 2022 [47].

**Table 3:** Comparison table of the PPG-based NIR BGL estimation.

Author (Reference)	Number of Features	Machine Learning Technique	R^2^
Monte-Moreno E. [38]	33	Random Forest	0.88
Habbu S. et al. [42]	28	Neural Networks	0.91
Yadav J. et al. [43]	17	Neural Networks	0.96
Hina A. et al. [45]	6	Fine Gaussian SVR	0.937
Hina A. et al. [47]	6	Ensemble Boosted Trees	0.956

## Data Availability

We have created our own dataset for this study. The research is still in progress so we cannot publish the dataset right now.
